# Supplementary material for: Excitonic topology and quantum geometry in organic semiconductors
Source: Nat Commun. 2025 May 19;16:4661. doi: 10.1038/s41467-025-59257-5 (PMC12089288; doi:10.1038/s41467-025-59257-5)
Supplement: Supplementary file 1 — Supplementary Information [file 41467_2025_59257_MOESM1_ESM.pdf]

Supplementary Information for "Excitonic topology and quantum geometry in organic semiconductors"

Wojciech J. Jankowski,[1, *] Joshua J.P. Thompson,[2] Bartomeu Monserrat,[1, 2] and Robert-Jan Slager[1, †]

[1] *TCM Group, Cavendish Laboratory, Department of Physics, J J Thomson Avenue, Cambridge CB3 0HE, United Kingdom*

[2] *Department of Materials Science and Metallurgy, University of Cambridge, 27 Charles Babbage Road, Cambridge CB3 0FS, United Kingdom*

(Dated: March 28, 2025)

In this Supplementary Information, we provide further details on:

Supplementary Note 1: Wannier centres and Berry phases

Supplementary Note 2: Decomposition of the excitonic Berry connection

Supplementary Note 3: Dualisation of the interacting Hamiltonian

Supplementary Note 4: Controlling excitonic topology with strain

Supplementary Note 5: Riemannian geometry of excitons

Supplementary Note 6: Derivation of the bound on excitonic spread

Supplementary Note 7: Numerical excitonic localisation

Supplementary Note 1: Wannier centres and Berry phases

We here briefly comment on the interplay of Berry phases and Wannier centres of electrons, holes, and excitons. In the context of electron and hole Wannier centres, we can recapture these in terms of corresponding Wannier centres, $\bar{w}_e$ and $\bar{w}_h$. Here, the electron/hole Wannier centres are defined as [1]:

$$\bar{w}_{e/h} = \left\langle w_0^{e/h} \middle| r_{e/h} \middle| w_0^{e/h} \right\rangle, \tag{1}$$

with $r_{e/h}$ the electron/hole position operator, and the corresponding Wannier states $\left| w_0^{e/h} \right\rangle$ obtained by Fourier-transforming electron and hole bands [1]:

$$\left| w_0^{e/h} \right\rangle = \frac{a}{2\pi} \int_{\mathrm{BZ}} \mathrm{d}k \; e^{\mathrm{i}k r_{e/h}} \left| u_k^{e/h} \right\rangle, \tag{2}$$

over the momentum space coordinates k in the first Brilouin zone (BZ). On the other hand, the excitonic Wannier centres $\bar{w}_{\mathrm{exc}}$:

$$\bar{w}_{\mathrm{exc}} = \langle w_0^{\mathrm{exc}} | \, R \, | w_0^{\mathrm{exc}} \rangle, \tag{3}$$

are defined with the exciton centre-of-mass position operator R and exciton Wannier states $|w_0^{\mathrm{exc}}\rangle$:

$$|w_0^{\mathrm{exc}}\rangle = \frac{a}{2\pi} \int_{\mathrm{BZ}} \mathrm{d}Q \; e^{\mathrm{i}QR} \, |u_Q^{\mathrm{exc}}\rangle, \tag{4}$$

which are also pictorially represented in Fig. 3 of the main text. Additionally, the relation between the Berry phases and the Wannier centres gives $\phi_{e/h} = 2\pi \bar{w}_{e/h}/a$ [1], which obtains the Berry phases for electron and holes. Analogously, the Berry phase of the excitons is equivalent to the shift of an excitonic Wannier centre $\phi_{\mathrm{exc}} = 2\pi \bar{w}_{\mathrm{exc}}/a$.

* wjj25@cam.ac.uk;
† rjs269@cam.ac.uk

Supplementary Note 2: Decomposition of the excitonic Berry connection

To compute the excitonic invariant P_{exc}, we utilise Eq. (3) of the main text, and decompose the excitonic Berry connection of the Eq. (2) of the main text into an envelope part and a single-particle part as:

$$A_{\text{exc}}(Q) = A^{\text{en}}_{\text{exc}}(Q) + A^{\text{sp}}_{\text{exc}}(Q). \tag{5}$$

The envelope part reads:

$$A^{\text{en}}_{\text{exc}}(Q) = \mathrm{i}\sum_k \psi^*_Q(k)\partial_Q\psi_Q(k), \tag{6}$$

and the single-particle part consists of the electron and hole contributions [2]:

$$A^{\text{sp}}_{\text{exc}}(Q) = \frac{1}{2}\sum_k |\psi_Q(k)|^2 \left[A^{\text{e}}(k+Q/2) + A^{\text{h}}(k-Q/2)\right]. \tag{7}$$

In turn, the single-particle electron and hole Berry connections are given by $A^{\text{e/h}}(k \pm Q/2) = \mathrm{i}\left\langle u^{\text{e/h}}_{k\pm Q/2}\middle|\partial_Q u^{\text{e/h}}_{k\pm Q/2}\right\rangle$.

We find that in the regimes realised by the organic materials in regions I and II of the phase diagram of Fig. 2 of the main text, the contribution to ϕ_{exc} due to the envelope part $A^{\text{en}}_{\text{exc}}(Q)$ vanishes, whereas the single-particle contribution $A^{\text{sp}}_{\text{exc}}(Q)$ due to the band geometry of the constituent electrons and holes (see "Riemannian geometry of excitons" below), gives $\phi_{\text{exc}} = \pi$ on integrating. Notably, the envelope contribution vanishes, as under the combination of inversion ($\mathcal{P}$) and time-reversal ($\mathcal{T}$) symmetries present in the considered materials (see also "Derivation of the bound on excitonic spread" below), the envelope function is a single-valued, *real* smooth scalar function, which unlike the electron Bloch bands, cannot yield any non-trivial boundary terms due to $U(1)$-phase factors, on integrating over BZ.

Moreover, beyond the numerical evaluation of the excitonic Berry phase and the dualisation argument detailed further below, we analytically model the topology of excitonic wavefunctions within the phase diagram by asserting an effective form of the excitonic envelope function. Explicitly, the exciton envelope function for the $t_2 > t_1$ regime, obtained by a qualitative fit to the Wannier equation solution, reads:

$$\psi_Q(k) = \mathcal{N}\Big[\cos(k+Q)e^{-(k+Q/2)^2/2} + \cos(k-Q)e^{-(k-Q/2)^2/2}\Big], \tag{8}$$

with the normalisation constant $\mathcal{N}$. With this expression, we obtain a vanishing envelope contribution $\oint \mathrm{d}Q\ A^{\text{en}}_{\text{exc}}(Q) = 0$, confirming that the excitonic topology is determined by the topology of the underlying electron and hole states. Consistently, we show the smooth dependence of the excitonic envelope functions $\psi_Q(k)$ on the exciton momentum Q for even and odd excitonic bands in Supplementary Fig. 1.

Supplementary Note 3: Dualisation of the interacting Hamiltonian

To reflect the physics of the (screened) density-density interactions, we truncate the long-range interaction expansion at the level of the nearest-neighbor interactions $U_1 \equiv U^{AB}_1$ and $U_2 \equiv U^{AB}_2$ [3], assuming that $U_1, U_2 \gg U_{i>2}$. In the limit $t_1, t_2 \gg U_1, U_2$, we obtain the standard SSH model for electronic bands, and $\oint \mathrm{d}Q\, A^{\text{en}}_{\text{exc}}(Q) = 0$. In this case, the entire contribution to excitonic topology can only emerge from the electrons and holes $\oint \mathrm{d}Q\ A^{\text{sp}}_{\text{exc}}(Q) = \pi$. Intuitively, this corresponds to the formation of excitons on the same site where the constituent electrons and holes reside.

In the opposite limit, $U_1, U_2 \gg t_1, t_2$, the Hamiltonian reduces to:

$$H = \frac{U_1}{2}\sum_j [n_{A,j}n_{B,j} + n_{B,j}n_{A,j}] + \frac{U_2}{2}\sum_j [n_{A,j+1}n_{B,j} + n_{B,j}n_{A,j+1}]. \tag{9}$$

Notably, we recognise that $n_{B,j} = c^\dagger_{B,j}c_{B,j} = c^\dagger_{B,j}h^\dagger_{B,j} \equiv b^\dagger_{B,j}$, where $h^\dagger_{B,j}$ is a creation operator for a hole, and in combination with the creation operator of an electron $c^\dagger_{B,j}$, gives a localised exciton creation operator $b^\dagger_{B,j}$. Similarly, for sites A we write: $n_{A,j} = c^\dagger_{A,j}c_{A,j} = h_{A,j}c_{A,j} \equiv b_{A,j}$. On relabelling $\frac{U_{1/2}}{2} \rightarrow -t^{\text{exc}}_{1/2}$, we obtain a dualised SSH Hamiltonian for excitons:

$$H = -t^{\text{exc}}_1 \sum_j b^\dagger_{B,j}b_{A,j} + \text{h.c.} - t^{\text{exc}}_2 \sum_j b^\dagger_{B+1,j}b_{A,j} + \text{h.c.}, \tag{10}$$

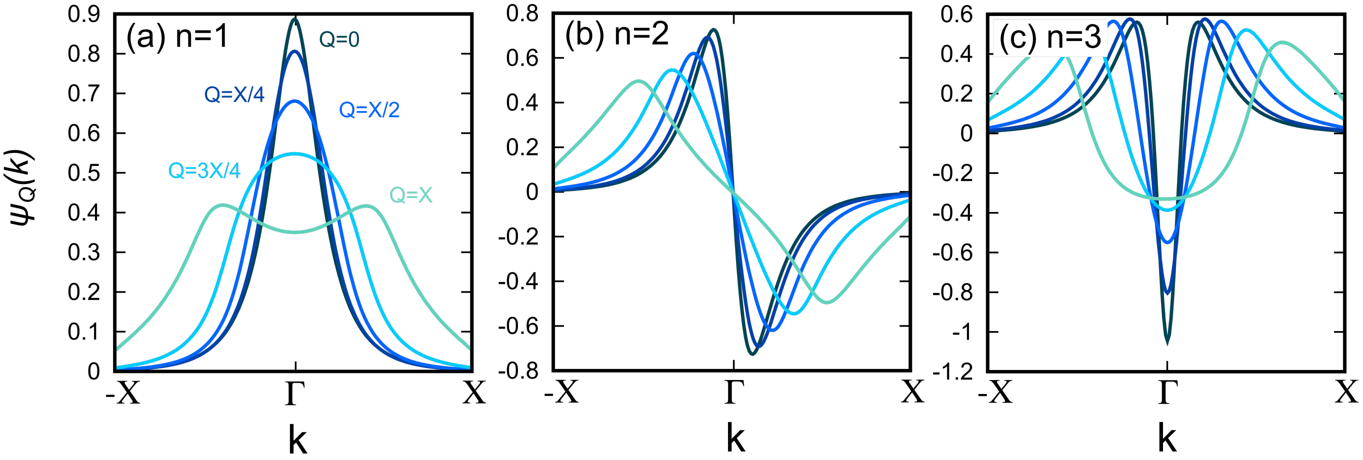

Supplementary FIG. 1. **Excitonic envelope wavefunctions.** **(a)** Excitonic envelope function for the first excitonic band ($n = 1$). **(b)** Excitonic envelope function for the second excitonic band ($n = 2$). **(c)** Excitonic envelope function for the third excitonic band ($n = 3$). The excitonic envelope functions $\psi_Q(k)$ are shown for different exciton centre-of-mass momenta Q within the momentum space range $(0, X)$. Here, $k = X$ is the momentum at the BZ edge and $k = \Gamma$ denotes the BZ centre. In the considered polyacenes, the excitonic envelope functions introduce no non-trivial excitonic Berry phase ϕ_{exc} contributions, with all the non-trivial topology of excitons being inherited from the topological electrons and holes.

where the Hubbard interactions between the electrons and holes on different sites effectively drive exciton hopping, or, in other words, exciton delocalisation. Notably, as $t^{\text{exc}}_{1/2}$ are not independent from the electronic $t_{1/2}$, we observe no regions where topological excitons would be obtained in the lowest excitonic band from trivial electrons and holes in [3] in the phase diagram, which would correspond to an additional topological regime, cf. Fig. 3 in the main text. The reason for the lack of such independence in the real material context (unlike in an abstract tight-binding model construction) is that both $t_{1/2}$ and $U_{1/2}$ are given by the matrix elements of the same Wannier states and local Hamiltonians, as well as (Coulomb) interaction potentials, and are therefore not independent. It should be noted here that the interactions manifestly do *not* break the inversion symmetry, by construction of the model.

Importantly, we stress that large dielectric screening ($\epsilon_s \gg 1$) can suppress the interaction strengths U_1, U_2. In the studied experimentally relevant organic systems, the dielectric screening allows to effectively control, i.e. suppress, the interaction strengths U_1, U_2. In that context, U_2 might be significantly lower than U_1 ($U_1 \gg U_2$), as long as $t_1 > t_2$, which imposes that the charge density over the bonds with enhanced hoppings is larger, consistently with the Wannier equation, and with the chemistry of the material [4]. At the DFT level, the Hartree term driven by the local mean-field electric charge density requires that $t_2 > t_1$ results in $U_2 > U_1$. It should be stressed that, on the contrary, the exchange interaction in the considered organic materials is negligible, therefore, the Hartree term dominates.

Supplementary Note 4: Controlling excitonic topology with strain

First principles results on the impact of strain on the structure and single-particle electronic band structure of the polyacenes are illustrated in Supplementary Fig. 2. In Supplementary Fig. 2(a), we show the change in the molecular polymer unit as a result of compressive (red) and tensile (green) strain. The impact of strain is primarily localised on the central carbon ring leading to a modulation of the C–C (carbon-to-carbon) distance, both along and perpendicular to the polymer chain.

The electronic bandstructure of strained polypentacene is shown in Supplementary Fig. 2(b), with both compressive and tensile strain. The VBM is set to 0 eV in all cases for comparison. For 5% compressive strain the bandwidth is reduced and the band gap increases while for 5% tensile strain the opposite is observed. This is in accord with the topological electrons and holes in polypentacene, as isolated molecules should always be topologically trivial, and large tensile strains should move the band structure towards that of a topologically trivial state. At 10% tensile strain the band structure is quantitatively similar to that of 5% strain. However, upon inspecting the electronic orbitals, Supplementary Fig. 2(c), a stark change in the orbital shape is seen at $k = X$ as compared to the unstrained molecule [c.f., Fig. 6(e)]. Interestingly, the overall shape and structure of these orbitals in the central carbon ring is close to those observed in polyanthracene showing that the topological electronic states in highly strained polypentacene have become trivialised. This is realised as the trivial exciton shown in Fig. 2(b) of the main text ($t_1 > t_2$).

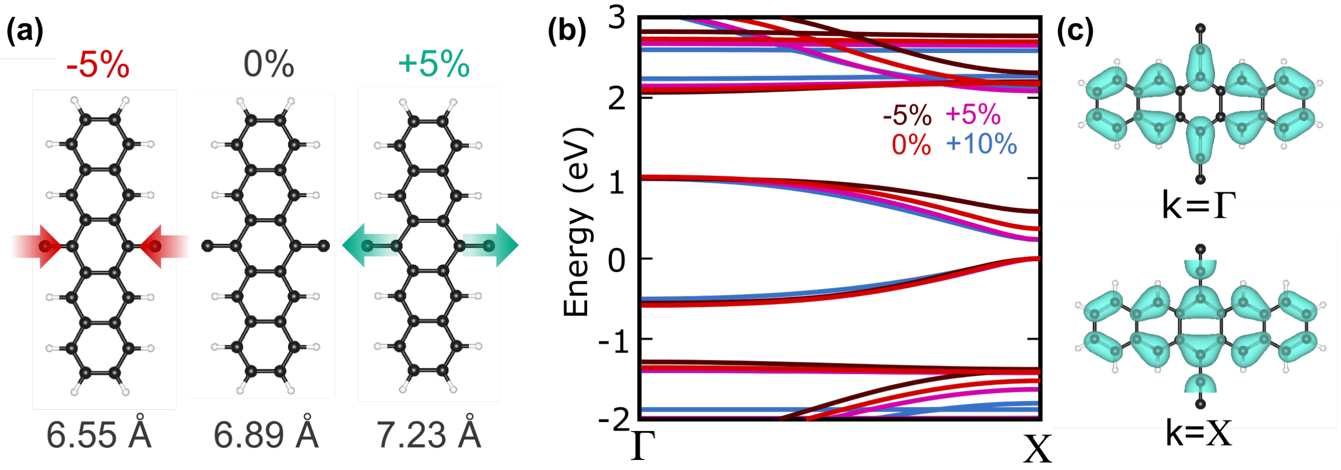

Supplementary FIG. 2. **First principles calculations and the impact of strain on polyacenes.** **(a)** Impact of uniaxial strain $|\gamma| \leq 10\%$ applied along the polymer chain axis on the crystal structure of polypentacene. The percentage values of the strain, γ, and the values of the corresponding lattice parameters, a', are provided above and below the strained molecular structures, respectively. **(b)** Electronic band structure of polypentacene at different strains: $\gamma = 0\%$ (red), $\gamma = -5\%$ (black), $\gamma = 5\%$ (purple), $\gamma = 10\%$ (blue) are shown. **(c)** Valence band electronic orbital at $k = \Gamma$ and $k = X$. The change in charge centre compared to the unstrained pentacene [c.f Fig. 6(e) of the main text] is characteristic of the polyanthracene [c.f Fig. 6(d) of the main text].

From an analytical perspective, we can model the effect of strain as renormalising the hopping parameters as,

$$t_{1/2}(\gamma) = t_{1/2}(\gamma = 0)e^{-\gamma C_{1/2}} \tag{11}$$

where $\gamma = \Delta a/a = (a' - a)/a$ is the uniaxial strain strength, captured by the change in the lattice parameter $\Delta a = a' - a$, that changes from the original value a to a'. $C_{1/2}$ are phenomenological constants capturing distinct scaling of $t_{1/2}$ under the action of strain. Importantly, $C_1 > C_2$ ($C_1 < C_2$) is a necessary condition for an excitonic topological phase transition to happen, if $t_1 > t_2$ ($t_1 < t_2$). Otherwise, the crossover of hoppings, which induces the topological transition, cannot occur. Notably, a similar crossover associated with an electronic strain-induced topological phase transition was recently observed and characterised in graphene nanoribbons [5].

Supplementary Note 5: Riemannian geometry of excitons

In this section, we further elaborate on the Riemannian structure of the Bloch bundles of the excitonic wavefunctions and the associated excitonic quantum metric. We start by recognizing that we can equip a set, or bundle, of excitonic states $|u^{\text{exc}}_{\mathbf{Q}}\rangle$ with a Hermitian metric, known otherwise as a quantum-geometric tensor (QGT). Explicitly, we write [6]:

$$Q^{\text{exc}}_{ij} = \langle \partial_{Q_i} u^{\text{exc}}_{\mathbf{Q}} | 1 - \hat{P} | \partial_{Q_j} u^{\text{exc}}_{\mathbf{Q}} \rangle , \tag{12}$$

where $\hat{P} = |u^{\text{exc}}_{\mathbf{Q}}\rangle \langle u^{\text{exc}}_{\mathbf{Q}}|$ is a projector onto the excitonic band of interest. The excitonic QGT can be decomposed in terms of Riemannian (real) and symplectic (imaginary) parts. The imaginary part defines an excitonic Berry curvature, which can be non-trivial only in two- or higher-dimensional systems, whereas the real part defines an excitonic Riemannian metric $g^{\text{exc}}_{ij} \equiv \mathfrak{Re}\, Q^{\text{exc}}_{ij}$, which can be non-vanishing even in one-dimensional systems.

The metric defined as the real part of the QGT explicitly reads:

$$g^{\text{exc}}_{ij} = \frac{1}{2}\Big[\langle \partial_{Q_i} u^{\text{exc}}_{\mathbf{Q}} | \hat{Q} | \partial_{Q_j} u^{\text{exc}}_{\mathbf{Q}} \rangle + \langle \partial_{Q_j} u^{\text{exc}}_{\mathbf{Q}} | \hat{Q} | \partial_{Q_i} u^{\text{exc}}_{\mathbf{Q}} \rangle \Big] , \tag{13}$$

where $\hat{Q} = 1 - \hat{P}$. In the one-dimensional case, which we consider in this work, there exists a single excitonic metric component of interest:

$$\begin{aligned} g^{\text{exc}}_{xx} &= \frac{1}{2}\Big[\langle \partial_{Q_x} u^{\text{exc}}_{Q_x} | \partial_{Q_x} u^{\text{exc}}_{Q_x} \rangle - \langle \partial_{Q_x} u^{\text{exc}}_{Q_x} | u^{\text{exc}}_{Q_x} \rangle \langle u^{\text{exc}}_{Q_x} | \partial_{Q_x} u^{\text{exc}}_{Q_x} \rangle + \text{c.c.} \Big] \\ &= \langle \partial_{Q_x} u^{\text{exc}}_{Q_x} | \partial_{Q_x} u^{\text{exc}}_{Q_x} \rangle - \langle \partial_{Q_x} u^{\text{exc}}_{Q_x} | u^{\text{exc}}_{Q_x} \rangle \langle u^{\text{exc}}_{Q_x} | \partial_{Q_x} u^{\text{exc}}_{Q_x} \rangle . \end{aligned} \tag{14}$$

We note that, on taking normalised eigenvectors $\langle u^{\rm exc}_{\mathbf{Q}}|u^{\rm exc}_{\mathbf{Q}}\rangle = 1$, and using the identity $0 = \partial_Q(\langle u^{\rm exc}_{\mathbf{Q}}|u^{\rm exc}_{\mathbf{Q}}\rangle) = \langle u^{\rm exc}_{\mathbf{Q}}|\partial_Q u^{\rm exc}_{\mathbf{Q}}\rangle + \langle \partial_Q u^{\rm exc}_{\mathbf{Q}}|u^{\rm exc}_{\mathbf{Q}}\rangle$, we obtain $\langle u^{\rm exc}_{\mathbf{Q}}|\partial_Q u^{\rm exc}_{\mathbf{Q}}\rangle = -\langle \partial_Q u^{\rm exc}_{\mathbf{Q}}|u^{\rm exc}_{\mathbf{Q}}\rangle$. Hence, in the one-dimensional case of interest, and taking $Q \equiv Q_x$, i.e. dropping x-index for simplicity, we can also write,

$$g^{\rm exc}_{xx} = \langle \partial_Q u^{\rm exc}_Q|\partial_Q u^{\rm exc}_Q\rangle - A_{\rm exc}A_{\rm exc}, \tag{15}$$

where $A_{\rm exc} = {\rm i}\langle u^{\rm exc}_Q|\partial_Q u^{\rm exc}_Q\rangle$.

In general, the exciton quantum metric can be decomposed as:

$$g^{\rm exc}_{ij}(\mathbf{Q}) = g^{\rm en}_{ij}(\mathbf{Q}) + g^{\rm sp\text{-}en}_{ij}(\mathbf{Q}) + g^{\rm sp}_{ij}(\mathbf{Q}), \tag{16}$$

with the envelope contribution:

$$g^{\rm en}_{ij}(\mathbf{Q}) = \frac{1}{2}\sum_{\mathbf{k}}[\partial_{Q_j}\psi^*_{\mathbf{Q}}(\mathbf{k})\partial_{Q_i}\psi_{\mathbf{Q}}(\mathbf{k}) + \partial_{Q_i}\psi^*_{\mathbf{Q}}(\mathbf{k})\partial_{Q_j}\psi_{\mathbf{Q}}(\mathbf{k})] - A^{{\rm en},i}_{\rm exc}A^{{\rm en},j}_{\rm exc}, \tag{17}$$

the single-particle contribution:

$$g^{\rm sp}_{ij}(\mathbf{Q}) = \frac{1}{4}\sum_{\mathbf{k}}|\psi_{\mathbf{Q}}(\mathbf{k})|^2\Big[g^{\rm e}_{ij}(\mathbf{k}+\mathbf{Q}/2) + g^{\rm h}_{ij}(\mathbf{k}-\mathbf{Q}/2)\Big] - A^{{\rm sp},i}_{\rm exc}A^{{\rm sp},j}_{\rm exc}, \tag{18}$$

and the mixed single-particle/envelope term:

$$g^{\rm sp\text{-}en}_{ij}(\mathbf{Q}) = \frac{1}{2}\sum_{\mathbf{k}}\Big[\partial_{Q_i}|\psi_{\mathbf{Q}}(\mathbf{k})|^2\partial_{Q_j}A^{{\rm sp},i}_{\rm exc} + \partial_{Q_i}|\psi_{\mathbf{Q}}(\mathbf{k})|^2\partial_{Q_i}A^{{\rm sp},j}_{\rm exc}\Big] - \Big[A^{{\rm sp},i}_{\rm exc}A^{j}_{\rm exc} + A^{i}_{\rm exc}A^{{\rm sp},j}_{\rm exc}\Big]. \tag{19}$$

We note that the last term in each of the excitonic metric decomposition terms is the corresponding contribution from the excitonic Berry connection term, i.e. the second term from the decomposition of Supplementary Eq. (15). In the above, $g^{\rm e/h}_{ij}(\mathbf{k})$ denote the individual single-particle quantum metrics of the electrons and holes. As mentioned in the main text, the envelope term $g^{\rm en}_{ij}$ becomes significantly enhanced in the higher excitonic bands, as it involves more pronounced variations of the corresponding excitonic envelope functions, see Fig. 5 in the main text for reference.

As a concluding remark, we note that the Riemannian geometry of the excitons can be controlled with the dielectric screening, see Supplementary Fig. 3.

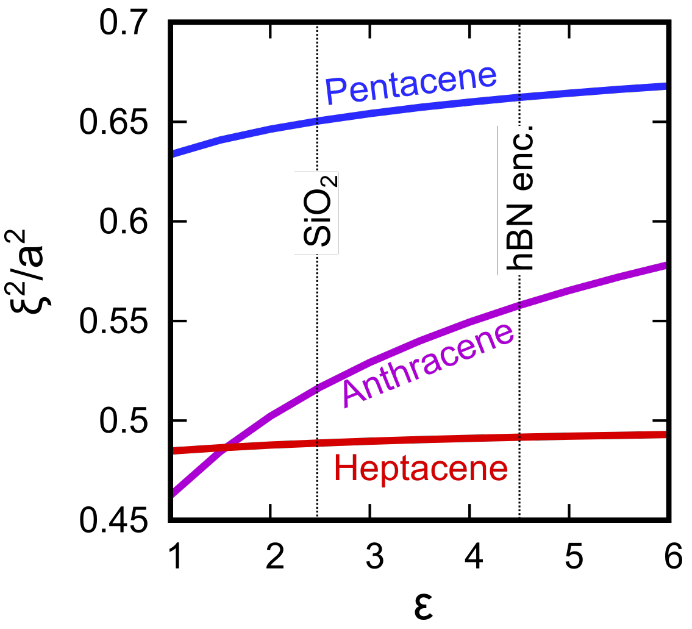

Supplementary FIG. 3. **Controlling excitonic geometry with dielectric environment**. Impact of background dielectric screening on the excitonic variance for polyanthracene (trivial, purple), polypentacene (topological, blue) and polyheptacene (red). Some typical dielectrics (SiO_2 substrate, hBN encapsulation) are marked with vertical lines.

While changing the dielectric screening does not result in any excitonic topological phase transitions, because of t_1 being correlated with U_1, and t_2 being correlated with U_2 within the studied materials, the geometry of the excitons can be controlled, while satisfying the topological bound. This offers for controlling the associated exciton transport features [7], which were studied in detail in the subsequent work.

Supplementary Note 6: Derivation of the bound on excitonic spread

The excitonic Riemannian metric encodes information about the localisation of the excitons, corresponding to the second moment, and in a one-dimensional context it is given by:

$$\xi^2 \equiv \mathrm{Var}R = \langle R^2 \rangle - \langle R \rangle^2 = \frac{a}{2\pi} \int_{\mathrm{BZ}} \mathrm{d}Q \; g^{\mathrm{exc}}_{xx}(Q), \tag{20}$$

where $\langle (\dots) \rangle = \langle w_0^{\mathrm{exc}} | (\dots) | w_0^{\mathrm{exc}} \rangle$ and $\left| w_j^{\mathrm{exc}} \right\rangle = \frac{a}{2\pi} \int_{\mathrm{BZ}} dQ \, e^{-\mathrm{i}Qja} \left| \psi_Q^{\mathrm{exc}} \right\rangle$ is an excitonic Wannier state with lattice vector $\mathbf{x} = ja$ labeling the unit cell of interest. Supplementary Eq. (20) follows by recognizing that Supplementary Eq. (14) implies:

$$\begin{aligned} g^{\mathrm{exc}}_{xx} &= \left\langle \partial_Q u_Q^{\mathrm{exc}} \middle| \partial_Q u_Q^{\mathrm{exc}} \right\rangle - \left\langle \partial_Q u_Q^{\mathrm{exc}} \middle| u_Q^{\mathrm{exc}} \right\rangle \left\langle u_Q^{\mathrm{exc}} \middle| \partial_Q u_Q^{\mathrm{exc}} \right\rangle \\ &= \sum_m \left\langle \partial_Q u_Q^{\mathrm{exc}} \middle| u_{mQ}^{\mathrm{exc}} \right\rangle \left\langle u_{mQ}^{\mathrm{exc}} \middle| \partial_Q u_Q^{\mathrm{exc}} \right\rangle \\ &= \sum_m \left\langle \psi_Q^{\mathrm{exc}} \right| R \left| \psi_{mQ}^{\mathrm{exc}} \right\rangle \left\langle \psi_{mQ}^{\mathrm{exc}} \right| R \left| \psi_Q^{\mathrm{exc}} \right\rangle \\ &= \left\langle \psi_Q^{\mathrm{exc}} \right| R (1 - \left| \psi_Q^{\mathrm{exc}} \right\rangle \left\langle \psi_Q^{\mathrm{exc}} \right|) R \left| \psi_Q^{\mathrm{exc}} \right\rangle . \end{aligned}$$

This result follows by recognizing that for $n \neq m$, we have $\left\langle \psi_{nQ}^{\mathrm{exc}} \right| R \left| \psi_{mQ}^{\mathrm{exc}} \right\rangle = \mathrm{i} \left\langle u_{nQ}^{\mathrm{exc}} \middle| \partial_Q u_{mQ}^{\mathrm{exc}} \right\rangle$, with $\left| \psi_{mQ}^{\mathrm{exc}} \right\rangle = e^{\mathrm{i}QR} \left| u_{mQ}^{\mathrm{exc}} \right\rangle$, and where m runs over all excitonic band indices apart from the index of the band $\left| u_Q^{\mathrm{exc}} \right\rangle$ of interest. Moreover, we used the resolution of the identity $1 = \sum_m \left| \psi_{mQ}^{\mathrm{exc}} \right\rangle \left\langle \psi_{mQ}^{\mathrm{exc}} \right| + \left| \psi_Q^{\mathrm{exc}} \right\rangle \left\langle \psi_Q^{\mathrm{exc}} \right|$. On changing the basis according to $|w_0^{\mathrm{exc}}\rangle = \frac{a}{2\pi} \int_{\mathrm{BZ}} \mathrm{d}Q \; e^{iQR} \left| u_Q^{\mathrm{exc}} \right\rangle$, and evaluating the matrix elements with the position operator R, we obtain the following relationship between the excitonic quantum metric g^{exc}_{xx} and spread ξ^2:

$$\frac{a}{2\pi} \int_{\mathrm{BZ}} \mathrm{d}Q \; g^{\mathrm{exc}}_{xx} = \langle w_0^{\mathrm{exc}} | \, R^2 \, | w_0^{\mathrm{exc}} \rangle - \langle w_0^{\mathrm{exc}} | \, R \, | w_0^{\mathrm{exc}} \rangle^2 = \langle R^2 \rangle - \langle R \rangle^2 = \langle (R - \langle R \rangle)^2 \rangle = \mathrm{Var}R \equiv \xi^2. \tag{21}$$

We now derive the main bound on the excitonic spread, captured by the excitonic metric due to the excitonic invariant P_{exc}. The excitonic $\mathbb{Z}_2$ invariant under inversion ($\mathcal{P}$) symmetry, in the gauge admitting only $\phi_{\mathrm{exc}} = 0/\pi$ under the symmetry, can be written as:

$$P_{\mathrm{exc}} = \frac{1}{\pi} \int_0^{\pi/a} \mathrm{d}Q \; [A_{\mathrm{exc}}(Q) + A_{\mathrm{exc}}(-Q)] = \frac{1}{\pi} \int_0^{2\pi/a} \mathrm{d}Q \; A_{\mathrm{exc}}(Q). \tag{22}$$

Moreover, the invariant can be alternatively deduced from the high-symmetry points (HSPs) $Q = 0$ and $Q = \pi$, analogously to how the topological invariants can be deduced from time-reversal invariant momenta (TRIMs) in the electronic topological insulators [8]. Equipped with the parities of the excitonic band at the HSPs, δ_i, the excitonic invariant satisfies,

$$(-1)^{P_{\mathrm{exc}}} = \Pi_i \delta_i. \tag{23}$$

Hence, $P_{\mathrm{exc}} = 1$ manifestly requires different band parity eigenvalues at $Q = 0$ and $Q = \pi$. We note, that in the studied organic materials, the excitonic states also satisfy the bosonic spinless time-reversal symmetry ($\mathcal{T}^2 = 1$), culminating in the spinless spatiotemporal inversion symmetry, $(\mathcal{PT})^2 = 1$ [9]. Therefore, topologically, the P_{exc} invariant corresponds to the first Stiefel-Whitney characteristic class $w_1 \in \mathbb{Z}_2$ [9–11] of the (real) excitonic Bloch bundle, as we also discuss later. We now utilise the topological invariant associated with the mentioned characteristic class to demonstrate the quantum-geometric bound.

Correspondingly, we use a Cauchy-Schwarz inequality $\left| \int \mathrm{d}Q \; f_1(Q) f_2(Q) \right|^2 \leq \left(\int \mathrm{d}Q \; |f_1(Q)|^2 \right) \left(\int \mathrm{d}Q \; |f_2(Q)|^2 \right)$, with functions $f_1(Q) = 1$ and $f_2(Q) = A_{\mathrm{exc}}(Q)$,

$$P^2_{\mathrm{exc}} = \frac{1}{\pi^2} \left| \int_0^{2\pi/a} \mathrm{d}Q \; A_{\mathrm{exc}}(Q) \right|^2 \leq \frac{1}{\pi^2} \left(\int_0^{2\pi/a} \mathrm{d}Q' \right) \left(\int_0^{2\pi/a} \mathrm{d}Q \; \left| A_{\mathrm{exc}}(Q) A_{\mathrm{exc}}(Q) \right| \right) \leq \frac{2}{\pi a} \int_0^{2\pi/a} \mathrm{d}Q \; g^{\mathrm{exc}}_{xx}(Q), \tag{24}$$

where a is the lattice parameter. Here, we additionally recognise that in the maximally-smooth gauge extremising the overlaps of Bloch states between the neighbouring Q-points, $\left\langle u_Q^{\mathrm{exc}} \middle| u_{Q+\Delta Q}^{\mathrm{exc}} \right\rangle \approx 1 + \left\langle u_Q^{\mathrm{exc}} \middle| \partial_Q u_Q^{\mathrm{exc}} \right\rangle \Delta Q \to 1$, that obtains

the maximally-localised Wannier functions [12]: $\left|A_{\text{exc}}(Q)A_{\text{exc}}(Q)\right| = \langle \partial_Q u_Q^{\text{exc}} | \hat{P} | \partial_Q u_Q^{\text{exc}} \rangle \leq \langle \partial_Q u_Q^{\text{exc}} | \hat{Q} | \partial_Q u_Q^{\text{exc}} \rangle = g_{xx}^{\text{exc}}(Q)$, i.e. the overlap of $|u_Q^{\text{exc}}\rangle$ and $|\partial_Q u_Q^{\text{exc}}\rangle$ is locally minimised, and is smaller than the overlap of $|\partial_Q u_Q^{\text{exc}}\rangle$ with all the other excitonic bands $|u_{mQ}^{\text{exc}}\rangle$ combined, as we also retrieve numerically. Intuitively, it should be noted that in the limit of a real gauge admitted under $\mathcal{PT}$ symmetry, the former overlap approaches vanishingly small values locally, consistently with the metric evolving as $g_{xx}^{\text{exc}}(Q) = \langle \partial_Q u_Q^{\text{exc}} | \hat{Q} | \partial_Q u_Q^{\text{exc}} \rangle \to \langle \partial_Q u_Q^{\text{exc}} | \partial_Q u_Q^{\text{exc}} \rangle$. Finally, on rearranging the derived inequality Supplementary Eq. (24), we obtain,

$$\xi^2 \equiv \frac{a}{2\pi} \int_0^{2\pi/a} \mathrm{d}Q \; g_{xx}^{\text{exc}}(Q) \geq \frac{a^2 P_{\text{exc}}^2}{4}, \tag{25}$$

showing that the maximally-localised excitonic Wannier functions support standard deviation associated with the spread: $\xi \geq a/2$, in the non-trivial phase with $P_{\text{exc}} = 1$. Mathematically, the non-trivial topological invariant $P_{\text{exc}} = 1$ associated with the non-trivial first Stiefel-Whitney class $w_1 \in \mathbb{Z}_2$ captures the orientability of a line subbundle of a real Bloch bundle [13], and is a global property of the excitonic Bloch bundles considered in this work. Intuitively, the non-trivial topology captured by the characteristic class implies the non-trivial quantum geometry locally, as a result of the associated non-orientability condition [10].

In the absence of non-trivial $P_{\text{exc}} \in \mathbb{Z}_2$, as well as of any other topological invariants in a considered phase, i.e. in the presence of only trivial excitons, any possible enhancement of the excitonic equivalent to the minimal spread of trivial states (ξ^2) is expected to be unlikely, as ξ^2 is unconstrained by any lower bound. This assertion, more formally yet intuitively, follows from the definition of the quantum metric:

$$g_{xx}^{\text{exc}}(Q) = \langle \partial_Q u_Q^{\text{exc}} | (1 - |u_Q^{\text{exc}}\rangle \langle u_Q^{\text{exc}}|) | \partial_Q u_Q^{\text{exc}} \rangle = \langle \partial_Q u_Q^{\text{exc}} | \hat{Q} | \partial_Q u_Q^{\text{exc}} \rangle = \langle \partial_Q u_Q^{\text{exc}} | \hat{Q}^2 | \partial_Q u_Q^{\text{exc}} \rangle = \left|\left| \hat{Q} | \partial_Q u_Q^{\text{exc}} \rangle \right|\right|^2, \tag{26}$$

where we employed a projector $\hat{Q} = 1 - |u_Q^{\text{exc}}\rangle \langle u_Q^{\text{exc}}|$ and used its idempotency, $\hat{Q} = \hat{Q}^2$, and Hermiticity $\hat{Q} = \hat{Q}^\dagger$ relations on connecting to the final norm $||...||$. In the case of the trivial topologies, the Bloch states $|u_Q^{\text{exc}}\rangle$ do not wind, hence $|\partial_Q u_Q^{\text{exc}}\rangle \to \mathbf{0}$, with $\mathbf{0}$, the norm-zero vector. Hence, $g_{xx}^{\text{exc}} = ||\hat{Q} |\partial_Q u_Q^{\text{exc}}\rangle ||^2 \to ||\hat{Q}\mathbf{0}||^2 \to 0$, as the Frobenius norm of $\hat{Q}$ is smaller than for the identity matrix 1 that sums over the projectors onto all bands, rather than onto a smaller number of bands, as $\hat{Q} = 1 - \hat{P}$, which on having subtracted $\hat{P} = |u_Q^{\text{exc}}\rangle \langle u_Q^{\text{exc}}|$, excludes the projection onto the original excitonic band $|u_Q^{\text{exc}}\rangle$.

Supplementary Note 7: Numerical excitonic localisation

Finally, in the light of the analytically derived quantum-geometric bound imposed by the non-trivial excitonic topology, we describe how the excitonic localisation§ that reflects the excitonic quantum geometry, can be retrieved numerically in a real material.

Here, we stress that to reflect the excitonic quantum geometry, the maximally-localised excitonic Wannier functions (MLXWFs) [14] need to be obtained, as the excitonic quantum metric reflects the *maximal* localisation as a gauge-invariant quantity. In an arbitrary gauge, the excitonic Wannier functions can take a less-localised form than fixed by the gauge-invariant quantum metric $g_{xx}^{\text{exc}}(Q)$. Contrary to the gauge-independent/gauge-invariant excitonic quantum metric and maximally-localised basis, an arbitrary excitonic Wannier function basis does not bear any physical meaning, as a gauge-dependent object. Such scenario is in a one-to-one correspondence analogy to the case of the electronic Wannier functions [1].

As a consequence, in the context of a real material, it is essential to numerically obtain the gauge-independent excitonic maximal localisation [14]. Following the approach of Ref. [14], this can be obtained by minimisation of a localisation functional F_{exc} that depends on the excitonic Wannier functions, $|w_0^{\text{exc}}\rangle$,

$$F_{\text{exc}} = \int_{-\infty}^{\infty} \mathrm{d}R \, \langle w_0^{\text{exc}} | R^2 | w_0^{\text{exc}} \rangle - \int_{-\infty}^{\infty} \mathrm{d}R \Big[\langle w_0^{\text{exc}} | R | w_0^{\text{exc}} \rangle \Big]^2. \tag{27}$$

For minimising the functional F_{exc}, the algorithm of WANNIER90 [15] can be directly utilised [14], on paralleling the standard method for minimising the spread of the electronic Wannier functions [1, 12, 16].

Consistently with the localisation procedure described in Ref. [12], the maximally-localised basis that minimises the localisation functional F_{exc}, can be numerically retrieved from sampling the possible $U(1)$ gauges $[U(Q) = e^{i\alpha(Q)}]$ of the isolated individual Fourier-transformed excitonic Bloch bands $|u_Q^{\text{exc}}\rangle$,

$$|w_j^{\text{exc}}\rangle = \frac{a}{2\pi} \int_{\text{BZ}} \mathrm{d}Q \; e^{-iQ(R)_j} U(Q) \, |u_Q^{\text{exc}}\rangle \,. \tag{28}$$

In the context of polyacenes, we further observe that such basis corresponds to the smoothest realisable waveform $w_0^{\mathrm{exc}}(R) = \langle R|w_0^{\mathrm{exc}}\rangle$, as quantified by the averaged analytic curvature of the excitonic Wannier function $\langle \partial_x^2 w_0^{\mathrm{exc}}(R)\rangle$.

Supplementary References

[1] Vanderbilt, D. *Berry phases in electronic structure theory: electric polarization, orbital magnetization and topological insulators* (Cambridge University Press, 2018). URL https://doi.org/10.1017/9781316662205.

[2] Kwan, Y. H., Hu, Y., Simon, S. H. & Parameswaran, S. A. Exciton band topology in spontaneous quantum anomalous Hall insulators: Applications to twisted bilayer graphene. *Phys. Rev. Lett.* **126**, 137601 (2021). URL https://link.aps.org/doi/10.1103/PhysRevLett.126.137601.

[3] Davenport, H., Knolle, J. & Schindler, F. Interaction-Induced Crystalline Topology of Excitons. *Phys. Rev. Lett.* **133**, 176601 (2024). URL https://link.aps.org/doi/10.1103/PhysRevLett.133.176601.

[4] Bradlyn, B. *et al.* Topological quantum chemistry. *Nature* **547**, 298 (2017). URL http://dx.doi.org/10.1038/nature23268.

[5] Tepliakov, N. V., Lischner, J., Kaxiras, E., Mostofi, A. A. & Pizzochero, M. Unveiling and manipulating hidden symmetries in graphene nanoribbons. *Phys. Rev. Lett.* **130**, 026401 (2023). URL https://link.aps.org/doi/10.1103/PhysRevLett.130.026401.

[6] Bouhon, A., Timmel, A. & Slager, R.-J. Quantum geometry beyond projective single bands (2023). 2303.02180.

[7] Thompson, J. J. P., Jankowski, W. J., Slager, R.-J. & Monserrat, B. Topologically-enhanced exciton transport (2024). URL https://arxiv.org/abs/2410.00967. 2410.00967.

[8] Fu, L. & Kane, C. L. Topological insulators with inversion symmetry. *Phys. Rev. B* **76**, 045302 (2007). URL https://link.aps.org/doi/10.1103/PhysRevB.76.045302.

[9] Bouhon, A., Bzdusek, T. & Slager, R.-J. Geometric approach to fragile topology beyond symmetry indicators. *Phys. Rev. B* **102**, 115135 (2020). URL https://link.aps.org/doi/10.1103/PhysRevB.102.115135.

[10] Nakahara, M. *Geometry, Topology and Physics.* LLC (Taylor & Francis Group, 2003).

[11] Ahn, J., Park, S., Kim, D., Kim, Y. & Yang, B.-J. Stiefel-Whitney classes and topological phases in band theory. *Chinese Physics B* **28**, 117101 (2019). URL http://dx.doi.org/10.1088/1674-1056/ab4d3b.

[12] Marzari, N., Mostofi, A. A., Yates, J. R., Souza, I. & Vanderbilt, D. Maximally localized Wannier functions: Theory and applications. *Rev. Mod. Phys.* **84**, 1419–1475 (2012). URL https://link.aps.org/doi/10.1103/RevModPhys.84.1419.

[13] Panati, G. Triviality of Bloch and Bloch–Dirac bundles. *Annales Henri Poincaré* **8**, 995–1011 (2007). URL https://doi.org/10.1007/s00023-007-0326-8.

[14] Haber, J. B., Qiu, D. Y., da Jornada, F. H. & Neaton, J. B. Maximally localized exciton Wannier functions for solids. *Phys. Rev. B* **108**, 125118 (2023). URL https://link.aps.org/doi/10.1103/PhysRevB.108.125118.

[15] Mostofi, A. A. *et al.* An updated version of Wannier90: A tool for obtaining maximally-localised Wannier functions. *Computer Physics Communications* **185**, 2309–2310 (2014). URL https://www.sciencedirect.com/science/article/pii/S001046551400157X.

[16] Marzari, N. & Vanderbilt, D. Maximally localized generalized Wannier functions for composite energy bands. *Phys. Rev. B* **56**, 12847–12865 (1997). URL https://link.aps.org/doi/10.1103/PhysRevB.56.12847.
