# Supplementary material for: Excitonic topology and quantum geometry in organic semiconductors
Source: Nat Commun. 2025 May 19;16:4661. doi: 10.1038/s41467-025-59257-5 (PMC12089288; doi:10.1038/s41467-025-59257-5)
Supplement: Supplementary file 2 — Description of Additional Supplementary Files [file 41467_2025_59257_MOESM2_ESM.pdf]

Description of Additional Supplementary Files

File name: Supplementary Data 1
Description: The atomic coordinates of the optimized computational models.
